# MicroRNAs as Active Players in the Pathogenesis of Multiple Sclerosis

**DOI:** 10.3390/ijms131013227

**Published:** 2012-10-15

**Authors:** Chiara Fenoglio, Elisa Ridolfi, Daniela Galimberti, Elio Scarpini

**Affiliations:** Department of Pathophysiology and transplantation, “Dino Ferrari” Center, University of Milan, IRCCS Foundation Cà Granda, Ospedale Maggiore Policlinico, 20122 Milan, Italy; E-Mails: ridolfi.elisa2@gmail.com (E.R.); daniela.galimberti@unimi.it (D.G.); elio.scarpini@unimi.it (E.S.)

**Keywords:** multiple sclerosis, microRNA, immune system, biomarker, central nervous system

## Abstract

MicroRNAs (miRNAs) are a recently discovered group of small noncoding RNAs that regulate gene expression post-transcriptionally. They are highly expressed in cells of the immune system, as well as in the central nervous system, and they are deregulated in various neurological disorders. Emerging evidence underlines an involvement of miRNAs in the pathogenesis of Multiple Sclerosis (MS). A number of miRNAs have been found to be dysregulated in blood cells from MS patients, in brain lesions, as well as in biological fluids such as serum and plasma. Despite miRNA altered expression likely showing a high tissue specificity, some profile similarities could be observed for certain miRNAs such as miR-326—such as upregulation in both active lesions and blood—though not for others such as miR-323, which demonstrated upregulation in whole blood, active brain lesions, and T-reg cells, but not in the serum of MS patients. In this review, the possible role of miRNAs in MS pathogenesis will be discussed according to all the available literature, with a particular emphasis on the possibility of considering extracellular miRNAs as a new source for both biomarker identification and therapeutic target discovery.

## 1. Introduction

**Figure 1:**
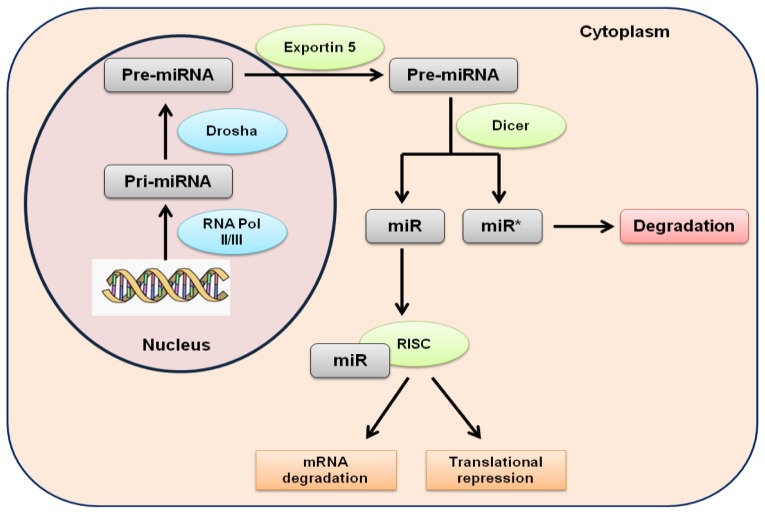
Biogenesis of human miRNAs. miRNAs are transcribed by RNA polymerase II and III in pri-miRNAs. pri-miRNAs are processed into pre-miRNAs by Drosha in the nucleus. Exportin 5 transports pre-miRNAs in the cytoplasm, where they are further processed by Dicer into mature double stranded miRNAs. One strand is incorporated in the RISC complex and the other is degraded. In this complex, miRNAs regulate gene expression.

MicroRNAs (miRNAs) are a class of small noncoding RNAs which have recently been discovered to be regulatory modulators of gene expression post-transcriptionally, either by targeting mRNA degradation or by inhibition of protein translation [1]. miRNAs directly modulate the expression of regulatory proteins that are required for normal development and function of the immune system. miRNAs have been estimated to roughly target 33% of human genes, highlighting their importance in gene regulation. miRNAs are expressed as 21–23 nucleotide RNA molecules initially transcribed by RNA polymerase II and III as long primary miRNAs (pri-miRNAs). Pri-miRNAs are processed in the nucleus into one or more precursor miRNAs (pre-miRNAs) by the enzyme Drosha. Pre-miRNAs are exported in the cytoplasm by exportin-5 and further processed by another enzyme Dicer into mature miRNAs, which is double stranded (miRNA duplex). The miRNA duplex is unwound and a strand (guide strand) is incorporated into the RNA-induced silencing complex (RISC), which contains another core component, Argonaute protein, while the other strand is degraded. In this complex, miRNAs lead to gene expression downregulation through two mechanisms: translational inhibition and target mRNA cleavage [1,2] (Figure 1). It has been shown that an individual miRNA is able to control the expression of more than one target mRNA and that each mRNA may be regulated by multiple miRNAs.

miRNAs play important roles in various biologic processes such as cell proliferation, development, differentiation, metabolism, apoptosis, angiogenesis, inflammation and immunity [3]. Aberrant miRNAs’ expression and function are associated with several human diseases, including cancer, neurodegeneration and autoimmunity [2,4,5].

Multiple sclerosis (MS) is the most common autoimmune disease of the central nervous system (CNS) among young adults. Its pathogenesis is only partly understood. It is believed that the disease process starts with increased migration of autoreactive lymphocytes across the blood–brain barrier (BBB), leading to axonal demyelination of neurons (lesions). Autoreactive T cell-mediated autoimmune response to myelin antigens causes inflammation, which in turn may lead to axonal degeneration contributing to the disability of patients with MS [6,7]. The cause of MS is not clear, but according to current data, the disease develops in genetically susceptible individuals with the contributions of environmental factors, such as infection, sunlight and vitamin D [8]. The clinical course of MS is extremely heterogeneous. The most common form is relapsing–remitting MS (RRMS), which affects more than 80% of MS patients. It is characterized by relapses of neurological dysfunction followed by periods of remission, in which symptoms improve or disappear. Over time, RRMS may develop into secondary progressive MS (SPMS) with slowly progressive neurological decline. Approximately, 15% of MS patients exhibit a more progressive disease without remission, namely, primary progressive MS (PPMS) [9].

The knowledge about how the immune system in MS patients is controlled differently than in unaffected individuals is still in its infancy. Thus, it is very useful to understand the functional significance of miRNAs with respect to MS pathogenesis. To this aim, this review, basing its proposals on the currently available data, will focus on the potential roles of miRNAs in MS, paying particular interest to the specific blood and brain lesions miRNA profiles and on their potential for the development of new drugs.

## 2. miRNAs and the Immune System

miRNAs, which control several aspects of immunity from the development and function of granulocytes, monocytes, macrophages, dendritic and natural killer cells [2], to the differentiation and activation of T and B cells [10], are also involved in MS pathogenesis.

The pioneer study about the role of miRNAs and T cells in MS was described in the paper by the group of Du *et al.* [11]. They identified miR-326 to be associated with interleukin-17 (IL-17) producing T-helper CD4^+^ cells (Th-17 cells), which are a subset of the effector helper T cells necessary for clearing foreign pathogens and are involved in the pathogenesis of chronic autoimmune diseases, including MS [12]. They demonstrated that miR-326 was over-expressed in Th-17 cells of patients with RRMS and promoted Th-17 differentiation, inhibiting Ets-1, a negative regulator of Th-17 differentiation [12].

Lindberg *et al.* [13] analyzed the expression of 365 miRNAs in CD4^+^, CD8^+^ T cells and B cells of peripheral blood of RRMS patients compared with healthy volunteers. Among the miRNAs considered, miR-17-5p, miR-92, miR-193a and miR-497 were found to be dysregulated in MS patients. In particular, miR-17-5p was upregulated in CD4^+^ cells from MS patients. miR-17-5p belongs to the miR-17-92 cluster that have roles in the development of autoimmune and lymphoproliferative diseases in mice [13]. miR-92 itself was found to be downregulated in B cells of patients with MS. A possible pathway, regulated by miR-17-92 cluster, is PI3K/Akt pathway, which regulates different stages of lymphocyte development, activation and survival [14]. miR-193a, which controlled the activation of caspase cascade [15], was dysregulated in CD4^+^ T cells in MS patients. Moreover, miR-497 was upregulated in CD4^+^ T cells and B cells, but was found to be downregulated in CD8^+^ T cells from MS patients versus controls. Very little is known about the function of miR-497 in autoimmune diseases or the immune system. Possible target genes could be cadherins, T cell activation and Wnt pathway genes, but none of these was experimentally validated [16].

De Santis *et al.* [17] performed a genome-wide expression analysis of miRNAs in regulatory T (Treg) CD4^+^ cells that lose their capacity to suppress the activation of the immune system and maintain homeostasis and tolerance to self-antigens in the course of MS [17]. Among the 723 human miRNAs tested, they found miR-106, miR-25, miR-19a and miR-19b significantly upregulated in Treg cells of MS patients versus controls. These miRNAs modulate the TGF-β signaling pathway, silencing the cell cycle inhibitor CDKN1A (p21) and the pro-apoptotic geneBCL2L11 (BIM) [18]. They speculated that the disruption of TGF-β pathway, involved in the maintenance of self-tolerance and T cell homeostasis, may be one way by which miRNA alteration promotes MS development [19].

In another study, miRNA profile of purified naive CD4^+^ T cells was analyzed. Authors focused their attention on this T cell subset in order to elucidate the mechanism by which CD4+ cell were induced to differentiate into pro-inflammatory phenotypes in MS patients. MiR-128 and miR-27b were increased in naive CD4^+^ T cells while miR-340 was increased in memory CD4^+^ T cells of patients with MS. Guerau-de-Arellano *et al.* [20] demonstrated that the dysregulated miRNAs could suppress the Th2 pathway through repression of BMI1 and IL-4 and their overexpression may mean a predisposition to the development of a Th1 response and autoimmunity in MS patients [20].

## 3. Blood and Brain Lesions miRNA Profile

Several studies performed miRNA profiling in MS and non-MS control subjects using peripheral blood mononuclear cells (PBMC) [21–23], whole blood [24,25], and brain lesions [26]. All reports showed altered miRNA expression profiles in MS patients compared to control subjects. Some discrepancies, however, were observed between the miRNAs that were identified as dysregulated in these studies. This could be partly attributed to differences in the studied material, or to differences in the miRNA level quantification methods (mainly qRT-PCR or microarray). The number of miRNAs analyzed appears very different according to the different studies. Moreover, patients under different treatment conditions were often included, and this could have influenced the results.

Otaegui *et al.* [21] examined the expression patterns of 364 miRNAs in PBMC from MS patients in the active phase of disease, in the remission phase, and in healthy controls. They found a specific miRNA signature of the relapse phase consisting in a strong dysregulation of miR-18b and miR-599, whereas a strong dysregulation of miR-96 levels was observed in the remission phase. Interestingly, the genes targeted by miR-96, are involved in immunological pathways such as the interleukin signaling pathway.

Fenoglio *et al*. [22] focused on immunologically relevant miRNAs, such as miR-21, miR-146a and -b, miR-150 and miR-155, and investigated their respective levels in PBMC from untreated MS patients compared with controls. A statistically significant increased expression of miR-21, miR-146a and -b was observed in RRMS patients as compared with controls. In contrast, no differences were found in the expression levels of both miR-150 and miR-155, highlighting the possibility of defining different disease entities with specific miRNA profiles.

Martinelli-Boneschi and [23] collaborators investigated the expression profile of 1145 miRNAs in PBMC from MS patients, some of them under treatment, and control subjects, finding a total of 104 dysregulated miRNAs in MS patients compared with controls. Best hits, let-7g and miR-150, were successfully replicated in a second independent population [23].

Keller *et al*. [24] investigated the expression levels of 866 miRNAs by using microarray analysis in peripheral blood samples of 20 patients with RRMS and 19 healthy controls. They identified 165 miRNAs significantly dysregulated in patients compared with controls. Further, they identified miR-145 as the best miRNA marker since it allowed the discrimination of MS from controls with a specificity of 89.5%, a sensitivity of 90%, and an accuracy of 89.7%. This study was one of the first to explore miRNA expression profile in blood as a biomarker for MS.

Cox *et al*. [25] performed a miRNA microarray analysis in peripheral blood samples of 59 untreated patients and 37 controls and found in the entire MS population a specific downregulation of miR-17 and miR-20a. Moreover, the same authors demonstrated that these miRNAs modulate T cell activation genes in a knock-in and knock-down T cell model and that the same T cell activation genes are also upregulated in MS, highlighting new approaches for therapy.

Specific miRNA profiles of both active and inactive MS lesions have been quantified in a seminal study performed by Junker *et al*. [26]. White matter lesions were obtained from human autopsy tissue and the expression levels of 365 miRNAs have been determined.

These authors found a specific miRNA signature in active or inactive brain lesions. In particular, miR-155, miR-326 and miR-34a were found to be upregulated in active MS lesions in comparison with inactive lesions or normal brain white matter. Interestingly, these miRNAs all target CD47, a regulatory membrane protein found to be downregulated in phagocitically active MS brain lesions [26].

## 4. Extracellular miRNA Profile

Cell-free miRNA can be detected in several human body fluids including plasma, serum, urine, and saliva [27,28]. Some miRNAs circulating in the blood have been identified as biomarkers in different human diseases such as cancer, cardiovascular diseases and brain injury [29,30] because they correlate with disease activity and prognosis, particularly in cancer [29,31]. Interestingly, circulating miRNAs are exceptionally stable in biological fluids, suggesting that miRNAs are released from cells in membrane-derived vesicles (exosomes) that protect them from blood RNase activity [29]. This evidence strongly suggests the utility of circulating miRNA as a potential clinical biomarker.

Until now, only one recent report has attempted to investigate extracellular miRNA levels in plasma samples from a cohort of MS and control subjects [32]. In particular, the authors carried out a microarray analysis of over 900 known miRNA transcripts from plasma samples collected from four MS individuals and their healthy controls matched to them in terms of gender and ethnicity.

Six out of 900 miRNAs tested were found to be significantly upregulated (miR-614, miR-572, miR-648, miR-1826, miR-422a and miR-22) and one plasma miRNA (miR-1979) significantly downregulated in MS patients.

Interestingly, both miR-422a and miR-22 have previously been implicated in MS [16,19,24,26]. In these previous studies, miR-422 was found to be upregulated in whole blood from RRMS samples compared to healthy controls [24] and downregulated in B-cell lymphocytes collected from RRMS patients compared to healthy controls [16]. Similarly, miR-22 expression was found to be increased in MS regulatory T cells, CD4 + CD25 + T cells, [19] and in MS active brain lesions, as well [26].

The reasons for these discrepancies could be related to the specific miRNA profile of the different tissues considered which could reflect the various biological effects for the transcripts in the cell type investigated [16].

Preliminary results from our laboratory examined a panel of 84 well characterized miRNAs in a serum from a cohort of MS samples compared with healthy controls and found significantly decreased expression levels of miR-15b, miR-23a and miR-223 in PPMS samples compared with controls [33].

Target prediction based upon TargetScan 6.1 (Whitehead Institute for Biomedical Research—MIT: Cambridge, MA, USA, 2012), www.microRNA.org and www.pictar.org websites led to the identification of several target genes of possible relevance to MS pathology. Both miR-15b and miR-23a target the *FGF-2* gene, a member of the fibroblast growth factor family. FGF-2 protein has been implicated in several biological processes, such as limb and nervous system development, wound healing, and tumor growth [34]. FGF-2 levels are reported to be elevated in the CSF of MS patients, particularly those with the active disease [35], and the gene was found to be differentially expressed in active and chronic MS lesions in post-mortem tissue [34], suggesting FGF-2 as a marker of inflammation in MS lesions.

Another interesting target gene of miR-15b is *KIF-1B* (Kinesin family member 1B), which encodes a motor protein that transports mitochondria and synaptic vesicle precursors. The *KIF*-1B gene was extensively investigated in the context of MS as a possible gene influencing MS susceptibility, though results remain controversial [36–42]. Among the genes targeted by miR-223 is the transcription factor mef-2c (myocyte enhancer factor 2C). Mutations and deletions at this locus have been associated with severe mental retardation and epilepsy [43].

miR-223 plays a role in the regulation of granulopoiesis by targeting mef-2c [36] and modulates the NF-κb pathway. Thus its downregulation could modulate immune inflammatory responses [44]. miR-223 was reported to be overexpressed in naive T cells of rheumatoid arthritis patients [45]. Several previous reports have shown a dysregulation of miR-223 in MS patients. Specifically, miR-223 was found to be upregulated in the blood [24], and in T regulatory cells from MS compared to healthy subjects and in active MS lesions compared to normal CNS areas in control subjects [26]. In contrast, in our preliminary study, we observed a downregulation of circulating miR-223 in the sera of MS subjects compared to controls. The reason for this discrepancy may be attributable to the cell-based versus circulating tissue tested.

Current knowledge of the biological significance of cell-free miRNA, especially in respect to intracellular miRNA, is still very limited. However, our preliminary findings could suggest that cellular miRNAs and miRNAs isolated from serum could be inversely correlated.

These are the very first investigations looking at the detection of miRNA levels in serum and plasma. These preliminary results suggest that circulating miRNAs could be of value in the research of novel biomarkers for MS.

## 5. miRNAs and Genetics

Genetic factors have long been demonstrated to play a role in disease susceptibility of MS. To date, the human leukocyte antigen (HLA) locus shows the strongest and most convincing association with MS susceptibility. The HLA genes are located in the major histocompatibility complex (MHC) region on chromosome 6p21.3. These genes encode highly polymorphic cell surface glycoproteins, which play roles in the self and nonself antigen recognition by the immune system [46]. In recent years, genome-wide association studies (GWAS) have identified 52 other MS risk loci, which are not associated with the HLA region. Most of these MS-associated loci are located close or inside genes, which have immunological functions [47].

Recently, several reports have shown that genetic alterations could influence miRNAs functions. Since miRNA biogenesis and target selection is highly sequence dependent, germline sequence variations (such as single nucleotide polymorphisms, SNPs) and post-transcriptional base modifications in either miRNA or miRNA-target site, could have profound effects on miRNA activity. These effects could be direct or indirect: direct effects are due to the presence of SNPs in the pri-miRNA, pre-miRNA or mature miRNA sequences and could possibly result in the impairment or enhancement on miRNA processing or function. Conversely, indirect effects involve SNPs in miRNA promoter sequences that could influence transcription or SNPs in mRNA sequences that create or destroy a target site [48]. Little is known about how genetics influences miRNA genes and consequently their roles in the pathogenesis of MS. There have been at least two studies that investigated the genetic association between miRNAs and MS [22,49].

Fenoglio *et al.* tested rs2910164, in the *miR-146a* gene, for association with MS [22]. miR-146a levels were found to be increased in other autoimmune diseases such as rheumatoid arthritis and psoriasis [50]. Moreover, miR-146a was overexpressed in regulatory T (Treg) cells, involved in the control of self-reactive T cells [51], a process which appears to be impaired in MS. They did not detect any differences considering both allelic and genotypic frequencies in patients versus controls. In addition, no differences were seen after stratifying for gender or disease subtype, however, the study was insufficiently powered to be definitive.

Paraboschi *et al.* [49] focused their attention on the role of miR-155 in MS susceptibility, genotyping four SNPs covering a genomic region of 19 kb located in close proximity to the miR-155 gene and its precursor BIC (the B-cell Integration Cluster). miR-155 is a key regulator in the development, maturation and function of different immune cells, such as Th1, Th2, B and Treg cells [2]. Murugaiyan *et al.* [52] demonstrated that miR-155 conferred susceptibility to experimental autoimmune encephalomyelitis, influencing both Th1 and Th17 effector subsets and contributing to autoimmune pathology [51]. Allele and genotypic frequencies between MS patients and controls were tested, but no statistically significant associations were found. However, SNP rs2829806 showed a weak trend towards a significant association. They also analyzed the haplotype frequency distributions in MS cases and controls, considering three adjacent SNPs across the region at a time. The GTT haplotype was over-represented in MS patients compared to controls, thus resulting associated with the disease status. This haplotype confers a 1.36 fold increased genetic risk of developing MS. Other two haplotypes, determined by the same polymorphisms, resulted significantly associated with the disease but they are quite rare in the analyzed population. However, the study did not reach the adequate power to demonstrate effective associations. Although further studies are needed to understand the functional effect of these variants on MS pathogenesis, these preliminary data show that SNPs affecting the expression of miR-155 may directly contribute to MS susceptibility [49].

## 6. MiRNA Therapeutic Potential

A novel and interesting approach in the development of a therapeutic strategy in the regulation of pathogenic gene expression is represented by the targeting of specific miRNAs. Recent findings suggest that it may eventually be possible to treat some neurological disorders by restoring or inhibiting miRNAs altered by disease pathology [52,53]. At present, one of the most promising methods of miRNA manipulation is represented by the use and delivery of modified oligonucleotides mimicking or inhibiting specific miRNA. Furthermore, the approach of using antisense oligonucleotides to bind and disrupt endogenous miRNAs, in some cases named “antagomirs” or “antiMirs”, has been used *in vivo* in several systems [54] for the repression of specific transcripts. A major issue in these efforts is to develop oligonucleotides able to be extremely efficient *in vivo* without significant toxicity. Locked nucleic acids (LNA) would be suitable to this purpose since they represent a family of conformationally locked nucleotide analogs that are relatively resistant to nuclease activity [55]. A relevant issue, however, remains the effective delivery of these molecules to the living organism, articularly when neurological disorders are considered, due to the relative difficulty in delivery modified oligonucleotides across the BBB. Recent efforts have also been directed toward developing small-molecule drugs able to influence miRNAs biogenesis or function [56]. Compounds able to disrupt miRNA biogenesis have recently been identified [57].

However, there is little evidence so far for the effects of the selective modulation of specific miRNAs in MS pathology [12,26].

As previously discussed, miR-326 levels have been found abundant in blood cells [12] and active lesions in patients with MS [26], promoting also the differentiation of Th17 cells that exert a pivotal role in antimicrobial defense at epithelial barriers and are also thought to be involved in MS pathogenesis [58,59]. Du *et al*. [12] recently observed that *in vivo* silencing of this miRNA in EAE resulted in a reduction in the number of Th17 cells and a less severe form of disease. Conversely, the same authors found that the overexpression of miR-326 led to an increase in the number of the Th17 cells and more severe EAE. Another interesting study by Murugaiyan *et al*. [26] focused on miR-155, which is induced in macrophages and dendritic cells after exposure to a variety of inflammatory cytokines such as INF-β, INF-γ and TNF-α and was already found to be elevated in MS brain lesions [26]. In this study Murugaiyan showed that miR-155 expression was increased in CD4^+^T cells in EAE and that miR-155^−/−^ mice had a delayed course and less severe disease with less inflammation in the CNS.

These authors underlined in their conclusion that the reduction of clinical severity in EAE by the administration of anti-miR 155 treatment was specifically observed early, before and after the observation of clinical symptoms, thus suggesting miR-155 as a new therapeutic target for intervention in MS. However, whether silencing of both miR-326 and miR-155 can be translated into humans for the treatment of MS still remains to be verified.

## 7. Treatment Effects on miRNA Profile

Some evidence recently arose about the influence of some common drugs used in MS on specific miRNA levels. In particular, two papers considered this interesting issue for the first time. The first study from Waschbisch *et al*. [60] analyzed the expression of selected miRNAs known to be involved in the regulation of the immune responses in 74 patients affected with RRMS and 32 healthy controls. Among patients, 36 subjects were treatment naive, the remaining treated with immunomodulatory drugs, 18 treated with INF-beta and 20 under Glatiramer Acetate (GA) treatment [60]. They found miR-326, miR-155, miR-146a and miR-142-3p expression levels dysregulated in PBMC from patients compared to controls and particularly decreased expression levels of miR-146a. miR-142-3p was observed in GA treated patients, whereas no difference was observed in INF-beta treated patients. Interestingly, the miRNAs found to be dysregulated in this study were already found to be involved in Th17 differentiation (miR-326 and miR-155), in the regulation of the immune tolerance (miR-142.3p and miR146a) and in the innate immunity (miR-146a). According to these results, GA treatment seemed to restore the levels of miR-142-3p and miR-146a. The second study was recently performed by Sieves *et al*. and compared the expression of 1059 miRNAs in B lympocytes from 10 untreated and 10 natalizumab-treated RRMS patients and 10 healthy controls [61].

Forty-nine miRNAs appeared to be downregulated in untreated MS patients compared with controls. In particular, they found a distinct signature of 10 differentially expressed miRNAs in natalizumab-treated patients compared with untreated patients. The most dysregulated were two clusters: miR-106b-25 and miR-17-92. Further, miRNA-mRNA interaction analysis performed revealed B cell receptor, phosphatidyl-inositol-3-kinase (PI3K) and phosphatase and tensin homology (PTEN), signaling as the key affected pathways.

## 8. Conclusions

The recent discovery of an involvement of microRNAs in MS opens a new field in the research of new therapeutic targets. After an initial poor overlap between results from the very first studies, recent studies suggest a role for specific miRNA, such as miR-326, miR-155 and miR-223, in MS pathogenesis.

Preliminary studies have started to analyze the possible genetic contribution of miRNA loci variability in MS, suggesting that the research on miRNAs has finally begun to be approached in a more comprehensive and definitive manner.

Dysregulated miRNA levels in biological fluids, such as plasma, serum or blood, could represent a new source of biomarkers in MS that could be helpful for disease prognosis and for discrimination of clinical subtype, thereby aiding therapeutic decisions or the monitoring of therapeutic effects. The discovery of MS biomarkers should greatly improve the diagnosis and management of MS and, in this context, miRNAs could have great value for the research of new therapeutic targets.
